# Applications of machine and deep learning to patient‐specific IMRT/VMAT quality assurance

**DOI:** 10.1002/acm2.13375

**Published:** 2021-08-03

**Authors:** Alexander F. I. Osman, Nabil M. Maalej

**Affiliations:** ^1^ Department of Medical Physics Al‐Neelain University Khartoum Sudan; ^2^ Department of Physics Khalifa University Abu Dhabi UAE

**Keywords:** deep learning, gamma passing rate prediction, IMRT quality assurance, machine learning, patient‐specific QA, radiation therapy, VMAT quality assurance

## Abstract

In order to deliver accurate and safe treatment to cancer patients in radiation therapy using advanced techniques such as intensity modulated radiation therapy (IMRT) and volumetric‐arc radiation therapy (VMAT), patient specific quality assurance (QA) should be performed before treatment. IMRT/VMAT dose measurements in a phantom using various devices have been clinically adopted as standard method for QA. This approach allows the verification of the accuracy of the dose calculation, data transfer, and the delivery system. However, patient‐specific QA procedures are expensive and require significant time and effort by the physicists. Over the past 5 years, machine learning (ML) and deep learning (DL) algorithms for predictions of IMRT/VMAT QA outcome have been investigated. Various ML and DL models have shown promising prediction accuracy and a high potential as time‐efficient virtual QA tool. In this paper, we review the ML and DL based models that were developed for patient specific IMRT and VMAT QA outcome predictions from algorithmic and clinical applicability perspectives. We focus on comparing the algorithms, the dataset sizes, the input parameters and features, the QA outcome prediction approaches, the validation, the performance, the clinical applicability, and the potential clinical impact. In addition, we discuss the present challenges as well as the future directions in the implementation of these models. To the best of our knowledge, this is the first review on the application of ML and DL based models in IMRT/VMAT QA predictions.

## 1 INTRODUCTION

Radiation therapy is widely adopted as an effective cancer treatment technique. Advanced radiotherapy techniques, such as intensity‐modulated radiation therapy (IMRT) and volumetric arc radiation therapy (VMAT), offer high dose conformity and sub‐millimeter spatial precision. These highly precise conformal methods allow physicians to maximize the tumor control probability while minimizing the normal tissue complication probability. However, the complex nature of these treatment techniques requires frequent patient‐specific quality assurance (QA) prior the treatment.^1^, ^2^, ^3^

Patient‐specific QA measurement procedure is recommended by AAPM TG 119 and 218^1^, ^3^ for IMRT and VMAT treatment to ensure that the treatments can be delivered as intended. Typically, this involves creating a QA plan by recalculating the patient treatment plan dose distribution on a suitable phantom. Then, measuring the dose distribution with a suitable device such as film, ion chamber or diode array detector, or electronic portal imaging device (EPID).^4^ Comparisons between measured and planned dose distributions are commonly quantified by means of a gamma analysis.^5^ This metric is routinely used for producing a quantitative measure based on dose and spatial criteria. The widely applied tolerance and action levels for gamma analysis are 3% dose difference and 3 mm distance‐to‐agreement, as recommended by AAPM TG 119 and 120.^1^, ^4^ The main disadvantages of the patient‐specific QA procedure are that it is expensive, time‐consuming, and often difficult to identify and correct the cause(s) of failure.^6^ Alternatively, computational‐based (software‐based) QA approaches are more time‐efficient but are less widely accepted. For instance, machine log file analysis method is time‐efficient and enables verification of the data transfer integrity and delivery system accuracy based on information recorded by the linear accelerator (Linac) control system.^7^ However, this approach is regarded as lacking independency from the delivery system.^7^

Machine learning (ML) and deep learning (DL) algorithms are able to produce predictions on new data after being trained on a finite dataset. Over the past 5 years, ML and DL algorithms have been developed and studied for predicting IMRT/VMAT QA outcome. These algorithms have the potential of providing time‐efficient and automated virtual QA tools. The developed ML/DL models would significantly reduce the radiation therapy treatment workload. The earlier reviews in the literature presented general overviews on applications of ML/DL in radiation therapy QA.^8^, ^9^, ^10^ In this paper, we present a comprehensive overview of the conventional ML‐based and modern DL‐based models for patient‐specific IMRT/VMAT QA from algorithmic and clinical utility perspectives. We discuss and compare these methods based on the data samples size, the input features, the QA outcome prediction approach, the model's validation, performance, and their clinical applicability and potential impacts. We also discuss current challenges and future directions of implementation of these models.

## 2 SEARCH STRATEGY

**FIGURE 1:**
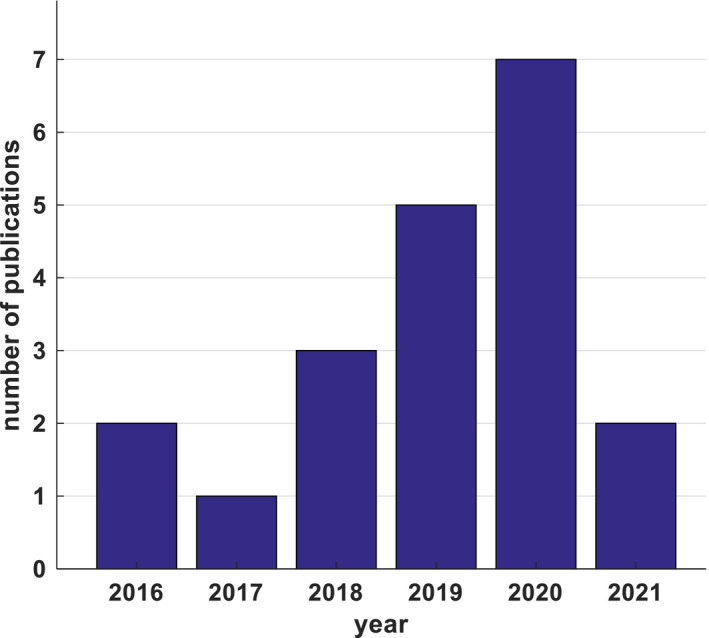
Yearly published articles related to intensity modulated radiation therapy and volumetric‐arc radiation therapy quality assurance outcome predictions using machine learning and deep learning algorithms, until March, 2021 (PubMed)

We performed a literature search on the web using the “PubMed” search engine. A total of 20 relevant published articles were found and were included in this review (Figure 1), with the most recent paper published in the first quarter of 2021 and the earliest one published in 2016. Figure 1 shows the number of yearly published articles for the last 5 years on applications of ML/DL algorithms in patient‐specific QA for IMRT and VMAT.

## 3 MACHINE LEARNING/DEEP LEARNING ALGORITHMS

In this section, we provide a brief overview of various ML and DL algorithms developed for QA outcome predictions in IMRT and VMAT without too much detail. Interested readers may refer back to the relevant references for more details.

### 3.1 Machine learning algorithms

Machine learning is a subset of artificial intelligence and it generally refers to set of algorithms that can learn to perform a specific task based on past data without explicit implementation of the solution.^11^ ML algorithms can be employed for regression or classification tasks. There are different types of ML algorithms and the commonly used ones relevant for this review are briefly described below.

#### 3.1.1 Regression algorithms

*Linear regression*^12^ is the simplest ML algorithms. It makes a prediction by computing the sum of the weights of input variables added to a bias term. During the training, the model searches for the set of parameters that make the best fit from inputs to output on the training data set. This is achieved by minimizing the mean squared error (MSE) loss function between the estimated output and the true value. In order to reduce the possibility of model overfitting, a regularization method such as the Least Absolute Shrinkage and Selection Operator (LASSO) regression, ridge regression, or elastic net is commonly applied.

*Poisson regression* is a form of generalized linear regression algorithm used to model count data. It is based on the assumption that the response variable has a Poisson distribution. Additionally, the logarithm of its expected value can be modeled by a linear combination of unknown parameters. This algorithm is trained in a same way as in linear regression. Poisson regression can also be regularized with LASSO regression which has an advantage of being capable to accommodate highly correlated set of features.^13^, ^14^

*Logistic regression*^15^ is a classification algorithm that is used to approximate discrete outcomes based on given independent variables. It applies a logistic function to model a binary dependent variable by estimating the parameters of a logistic model. These parameters are obtained during the training process. The logistic model determines the probability of a certain event (e.g. pass/fail) or occurrence of an event by fitting data to a logit function. Logistic regression is used when a binary outcome is needed. The weakness of this model lies in its instability when using data that are easily separable into certain classes or when using few data samples.

#### 3.1.2 Tree‐based algorithms

Tree‐based ML algorithms are a versatile type of supervised ML algorithms that can be used for regression and classification tasks. One of the most unique characteristics of these algorithms is that they exhibit high degree of interpretability of the predicted results. Consequently, we can understand the contribution of individual parameters to the prediction results as well as visualize how a prediction is made as the sample travels from the root to the leaf.

*Decision tree*^16^ is the simplest form of the tree‐based algorithms. It consists of several nodes and leaves. Each node represents a split of the training dataset based on one of the variables, whereas an individual leaf (decision) represents a target value of some of the samples. During the training, the model normally applies a recursively greedy training algorithm to search for the variables and the possible decision threshold for each node that optimally splits the training data into similar responses. This is practically solved by minimizing a cost function such as MSE between the predicted output and the true value. The nonparametric nature of the decision tree model increases the possibility of the model overfitting the training data by creating complex trees, thus it requires applying an appropriate regularization technique. Hyper‐parameters that are adjusted during the training of a decision tree model include the maximum tree depth, the minimum number of samples required at each node, and the loss function used to measure the quality of a split.

*Random forest*^17^, ^18^ is the complex form of the decision tree algorithms. It is basically an ensemble of decision trees that combines the results from a set of weaker decision trees predictors, where random errors cancel each other out and correct decisions are reinforced. Each of the decision trees is assembled using a randomly selected subset of the given variables and training examples. The bagging technique is used where a collection of estimators is trained on different random subsets of the training set with replacement. This approach reduces the variance of the average prediction to yield a better model. The hyper‐parameters described for the decision trees are optimized along with the number of trees in the optimized forest, the minimum sample of splits, and minimum sample of leaves. The final output of the ensemble random forest regression model is the average of the decision trees' outputs. Whereas, the final output of an ensemble random forest classification model is the class that has the most votes by the individual decision tress. Using a large number of trees in a random forest improves the model predictions power but it becomes harder to interpret.

*AdaBoost boosting or “adaptive weighted boosting”*^19^ is a boosting algorithm that combines a series of weak learner decision trees to build a composite strong learner with revised sample weights. This process is usually sequential, where subsequent models are trained based on the errors of the preceding ones. The training of this model initially starts with fitting a base decision tree regression algorithm to the whole training dataset. Then the algorithm iteratively trains the regression algorithm on the same dataset while adjusting the weights of training samples that have large errors. The final prediction is an ensemble of all the sequential learners in which the higher performers will contribute more to the prediction. An example of hyper‐parameters that is tuned during the training is the number of trees.

*Gradient boosting*^20^ is another boosting algorithm which adjusts instance weights at every iteration, by fitting new predictors to the residual errors made by the preceding one. It uses decision trees as the base regression algorithm for training and optimization. In this ensemble approach, the nodes in every decision tree take a different subset of features for selecting the best split. As a result, the individual trees are different, therefore, they are able to capture different characteristics from the data. Moreover, each new tree takes into account the errors of the preceding ones. The hyper‐parameter that are optimized include the maximum number of estimators, the learning rate, the fraction of training samples for fitting the individual base predictors, the maximum tree depth, and the minimum number of samples required to split an internal node.

*XGBoost or “extreme gradient boosting”*^21^ is an improved version of the gradient boosting algorithms with the decision tree regression algorithms built sequentially to rectify the previous trees' errors. The model is assembled by combining a set of weak decision trees iteratively forward with gradient descent optimization. XGBoost is also called a regularized boosting technique which helps to reduce overfitting. It inherently has both linear model and the tree learning algorithm. This gives the model high predictive power which makes it more accurate than the gradient boosting algorithm. Examples of hyper‐parameters that are adjusted for optimal solutions include the number of trees and the maximum depth of a tree.

#### 3.1.3 Support vector machines

Support vector machines (SVMs)^22^, ^23^ are another type of versatile ML algorithms that could be used for linear or nonlinear classification and regression problems. The SVM classification algorithm constructs a single or multiple optimal hyperplane(s) in a high‐dimensional space that separates the data into two or multiple classes with the maximum‐margin. The data points closest to the decision surface are called support vectors. This characteristic makes the SVM superior for classification tasks since it maximizes the margin around the separating hyperplane(s). SVM regression is a nonparametric technique that fits the data. SVMs implement kernels such as linear, polynomial, Gaussian radial basis function, sigmoid kernels, and others to map input variables to higher dimensional spaces to facilitate nonlinear predictive modeling.

#### 3.1.4 K‐nearest neighbors algorithms

K‐nearest neighbors (KNN)^24^, ^25^ is also a versatile ML algorithm that has been used for classification and regression. A KNN algorithm is a non‐parametric ML method with the input consisting of the k closest training examples in feature space. It works by finding the distances between a query and all the examples in the data and selecting the specified number examples (*k*) closest to the query. Then the algorithm votes for the most frequent label, in the case of classification, or the average of the values of k nearest neighbors in the case of regression. KNN algorithm is simple and easy to implement and does not require developing a model or tuning several parameters. However, it is computationally expensive as the number of examples or independent variables increase.

#### 3.1.5 Discriminant analysis algorithms

Linear discriminant analysis^26^ is one of the simple ML algorithms that is frequently used for classification problems. It works by creating linear boundaries between classes, and it is a generalization of Fisher's linear discriminant to find a linear combination of features that separates data samples into two or more classes. The algorithm defines the distance of a sample from the center of a class and creates a new set of axes to place members of the same group closer and move the groups farther apart. These new axes are discriminant axes, or canonical variates, which are linear combinations of the original variables. Linear discriminant analysis algorithm is easy to implement and interpret the results. However, the linear decision boundaries may not perfectly separate the classes particularly in a high‐dimensional setting where there are too many parameters. Thus, it requires proper regularization.

#### 3.1.6 Artificial neural networks

Artificial neural network (ANN)^27^ is a type of ML algorithms that are inspired by biological neural networks of the human brain. ANN consists of an input layer, a single or multiple hidden layers, and an output layer. The neurons represent the basic unit of these layers. A single neuron has input signals, a linear operation, a non‐linear operation, called “activation function”, and output signals. ANNs are trained for optimal fitting of the inputs and outputs for a regression task. For classification problems, multilayer perceptrons with threshold activation function are usually used to train the network. The hyper‐parameters that are tuned during the training include the number of neurons in a hidden layer, the number of hidden layers, the type of activation function used in each layer, and the learning parameters weights and biases. ANN performance is not significantly affected when few units fail to respond to the network. On the other hand, ANNs are argued as “black box” models and provide very little insight and require a relatively large training dataset.

### 3.2 Deep learning algorithms

Deep learning is a subset of ML algorithms that applies large‐scale hierarchical models with multi‐layer architectures to generate full representation and learn complicated inherent patterns of the data.^28^ Unlike ML, DL automatically extracts features from the input images, therefore, more features can be considered in the prediction. DL algorithms often surpass the traditional ML models if well‐trained. However, they lack the interpretability and they require large data size for proper training. The latter problem is solved by training the model via a transfer learning method.^29^ In this method, a pre‐trained DL model on a certain task using large‐scale data is reused as the starting point for training another model for a second task using limited training data. The commonly applied DL models are those based on neural networks.

#### 3.2.1 Deep neural networks

Deep neural networks (DNNs) are commonly used DL models. When a large number of hidden layers is used, an ANN model becomes a DNN.^30^

*Fully‐connected DNNs* are a model in which all neurons in a layer are connected with all neurons in the adjacent layer using pair‐specific connections. Thus, this full connection approach results in a very large number of trainable network parameters. The disadvantage of fully connected DNNs is the need for large amount of data and computational resources.

*Convolutional neural networks* (CNNs)^31^, ^32^ are another type of DNNs that use convolution and pooling operations to connect adjacent layers. The convolutional computation convolves a kernel with the preceding layer's image data to produce feature images. The output results are then fed to the next layer. Pooling operations, such as max‐pooling or average‐pooling, are added after convolution to reduce the number of pixels of the feature images. The advantage of CNNs over fully connected DNNs is that they typically require considerably less network parameters.

## 4 APPLICATIONS OF ML/DL FOR PATIENT‐SPECIFIC IMRT/VMAT QA

Recently, high attention has been given to implementation of ML and DL in medical physics fields, particularly medical imaging and radiation therapy. This interest led researchers to study various ML and DL models for patient‐specific QA outcome predictions for IMRT and VMAT. In this section, we discuss different ML/DL models and compare the used data sizes, the predictive input features, the QA prediction approaches, the model performances, and their clinical applicability.

### 4.1 ML/DL model for IMRT QA

Several ML and DL algorithms have been applied for patient‐specific IMRT QA outcome predictions. Out 11 studies found in our literature search, five studies directly predicted the gamma passing rate results for IMRT patient‐specific QA. The remaining six studies focused on using ML/DL algorithms for detecting and identifying errors for IMRT patient‐specific QA. A summary of ML and DL algorithms, dataset size used for training and development of the model, anatomic treatment sites involved, type of QA outcome predictions, number of extracted input features, and the key findings for patient‐specific IMRT QA are presented in Table 1.

**TABLE 1:** Summary of the studies that applied ML and DL models for IMRT QA outcome predictions.

Author/Year	Dataset size	Anatomic site	Algorithm	QA outcome prediction	No. of input features	Key results
Valdes et al. 2016^33^	498 Plans	Multiple	Poisson regression	Gamma passing rate	78	Errors within 3% at 3%/3 mm criteria
Valdes et al. 2017^34^	637 Plans	Multiple	Poisson regression	Gamma passing rate	90	Errors within 3.5% at 3%/3 mm criteria
Tomori et al. 2018^35^	60 Plans	Prostate	CNN	Gamma passing rate	–	Errors within 1.10% at 3%/3 mm criteria
Interian et al. 2018^36^	498 Plans	Multiple	CNN	Gamma passing rate	–	MAE = 0.70% at 3%/3 mm criteria
Wootton et al. 2018^37^	186 Beams (558 images)	Multiple	Logistic regression	Errors detection	17	3 Classes: AUC = 0.74
Lam et al. 2019^38^	1497 Beams	Multiple	Tree‐based algorithms	Gamma passing rate	31	Errors within 3% for 98% of predictions at 2%/2 mm criteria
Nyflot et al. 2019^39^	186 Beams (558 images)	Multiple	SVM, ANN, Decision Tree, and KNN	Errors detection	145	3 Classes: Accuracy = 64.3% (SVM)
Ma et al. 2020^40^	180 Beams (1620 images)	Multiple	Linear discriminant analysis, SVMs, & random forest	Errors detection	276	4 Classes: AUC = 0.86 (linear SVM)
Osman et al. 2020^41^	10 Plans (360,800 data points)	Multiple	ANN	Errors detection	14	RMSE = 0.0097 mm
Sakai et al 2021^42^	38 Beams (152 errors plans)	Multiple	KNN, SVM, Logistic Regression, and Tree‐based Algorithms.	Errors detection	837	4 Classes: AUC = 1.00 for leaf transmission factor error, 1.00 for dosimetric leaf gap error, & 0.80 for leaf positional error vs. error‐free (SVM)
Chuang et al. 2021^43^	267 IMRT & VMAT Plans (10,584,120 data points)	Multiple	Linear regression and tree‐based algorithms	Errors detection	7	RMSE = 0.0085 mm (boosted tree model)

Abbreviations: ANN, artificial neural network; AUC, area under the receiver operating characteristics curve; CNN, convolutional neural network; KNN, K‐nearest neighbors; MAE, mean absolute error; RMSE, root mean squared error; SVM, support vector machine.

Valdes et al.^33^ built a Poisson regression ML model trained on complexity features of IMRT plans to predict the gamma passing rate results at 3%/3 criteria measured with two‐dimensional (2D) array detector. The results showed that the gamma passing rate results could be predicted within 3% accuracy on a single institution dataset. The same research group^34^ extended their work to examine the generalizability of their previously developed Poisson regression on another institution dataset with patient‐specific QA measurement performed using EPID. The results revealed the generalizability of the Poisson regression with gamma passing rate prediction within 3.5% accuracy. Lam et al.^38^ assessed tree‐based (AdaBoost, random forest, and XGBoost) ML models for gamma passing predictions with patient‐specific QA measurements performed using EPID. Random forest and AdaBoost models exhibited the best performance, with gamma passing rate prediction error less than 4% for 2%/2 mm gamma criteria.

Due to its remarkable performance in different fields, DL has also been applied for IMRT/VMAT QA outcome predictions. Tomori et al.^35^ developed a CNN model trained from scratch on features extracted form sagittal dose distributions for gamma passing rate predictions with patient‐specific QA measurements performed using radiochromic film. The results have shown prediction errors within 1.10% at 3%/3 mm gamma criteria. Finally, Interian et al.^36^ built an ensemble DL model of pre‐trained CNNs via transfer learning (VGG‐16 ImageNet model) for gamma passing rate predictions using radiomics features derived from fluence maps with measurements done using a 2D array detector. The model achieved prediction performance comparable to that obtained with Poisson regression developed by Valdes et al.^33^ for 3%/3 mm gamma criteria.

Wootton et al.^37^ used a logistic regression ML model to detect random and systematic multileaf collimator (MLC) positional errors using radiomics features extracted from gamma maps measured using EPID. The model output is classified in three classes: (1) free‐error for gamma passing rate >95%; (2) random MLC positional error with an offset of each leaf by a distance between 0 and 2 mm; and (3) systematic MLC positional error with offset of all leaves by 2 mm in the same direction. The reported results showed a prediction accuracy with an area under the receiver operating characteristics curve (AUC) of 0.76 for random errors, 0.72 for systematic errors, and an overall AUC of 0.74 in detecting the three classes. This accuracy for detecting the MLC positional errors in IMRT patient‐specific QA was higher compared to the conventional gamma analysis.

In a similar study, Nyflot et al.^39^ evaluated a linear SVM, an ANN, a decision tree, and a KNN ML models trained on radiomics features in combination with CNN features derived from gamma maps to identify the errors for patient‐specific IMRT QA measurement using EPID. The SVM model achieved the best performance in predicting the three error classes with an accuracy of 0.64. Ma et al.^40^ assessed different ML models including linear discriminant analysis, linear SVM, radial basis function SVM, and random forest trained on structural similarity sub‐index maps that were calculated from portal dose images to predict the MLC positional errors and machine output variations in IMRT delivery. The authors simulated four types of machine errors (1) machine output or monitor unit (MU) variations; (2) random MLC errors; (3) same‐directional MLC shifts; and (4) opposite‐directional MLC shifts. The results indicated that linear‐SVM model achieved the best performance with an AUC of 0.86 in predicting the four error types, followed by linear discriminant analysis with AUC of 0.83, then random forest with an AUC of 0.80.

Sakai et al.^42^ studied decision tree, KNN, SVM, logistic regression, and random forest ML models to detect MLC positional errors using radiomics features derived from fluence maps measured with EPID. Four types of error classifications were used: (1) error‐free plan, in which the un‐modified beams were used; (2) transmission factor error plan, in which the value of the MLC transmission factor was altered by up to 20% of the original value of 0.01; (3) dosimetric leaf gap error plan, in which the value of the MLC dosimetric leaf gap was altered by up to 20% of the original value of 0.08 mm; and (4) MLC positional error, in which a leaf of one side‐bank was opened by up to 1.0 mm from the original position. The SVM model achieved the beast performance with AUC of 1.00, 1.00, and 0.80 in classifying the MLC transmission factor error, dosimetric leaf gap error, and MLC positional error respectively. Osman et al.^41^ built an ANN model to predict the MLC positional deviations during the dynamic IMRT treatment delivery using log file data. Their model revealed high prediction accuracy for all individual MLCs with a maximum root MSE (RMSE) of 0.0097 mm between the predicted and the actual leaf positions. Chuang et al.^43^ studied various regression ML models including simple and multivariate linear regressions, decision tree, and ensemble method (boosted tree and bagged tree model) using trajectory log files data to predict thee MLC positional errors for IMRT and VMAT treatment. The results showed that the boosted tree model had the best performance with RMSE of 0.0085 mm.

### 4.2 ML/DL model for VMAT

Applications of ML and DL algorithms for patient‐specific QA outcome predictions have also been extended to VMAT. A total of nine studies were found in our literature search. Six studies focused on predicting the gamma passing rate results for VMAT patient‐specific QA. The remaining three studies focused on errors detection and identification for VMAT patient‐specific QA. A summary of ML and DL algorithms, dataset size used for training and development the model, anatomic treatment sites involved, type of QA outcome predictions, number extracted input features, and the key findings for patient‐specific VMAT QA are presented in Table 2.

**TABLE 2:** Summary of the studies that applied ML and DL models for VMAT QA outcome predictions.

Author/Year	Dataset size	Anatomic site	Algorithm	QA outcome prediction	No. of input features	Key results
Carlson et al. 2016^44^	74 Plans (3,161,280 data points)	Multiple	Linear regression, random forest, and cubist	Errors detection	6	RMSE = 0.193 mm (linear regression)
Li et al. 2019^45^	303 Plans	Multiple	Poisson regression	Gamma passing rate	54	Errors within 3.5% for 90% of predictions at 3%/3 mm criteria
Ono et al. 2019^46^	600 Plans	Multiple	Regression tree analysis, multiple regression analysis, and ANN	Gamma passing rate	28	Mean prediction error = –0.2% (ANN) at 3%/3 mm criteria
Granville et al. 2019^47^	1620 Beams	Multiple	SVM	Errors detection	60	3 Classes: AUC = 0.88 (macro‐averaged)
Wall and Fontenot 2020^48^	500 Plans	Multiple	Linear regressions, SVM, tree‐based algorithms, and ANN	Gamma passing rate	241	MAE = 3.75% (SVM) at 3%/3 mm criteria.
Hirashima et al. 2020^49^	1255 Plans	Multiple	Tree‐based algorithms	Gamma passing rate	875	MAE = 4.2% & AUC = 0.83 at 2%/2 mm criteria
Tomori et al. 2020^50^	147 Plans	Multiple	CNN	Gamma passing rate	–	MAE = 0.63% at 3%/3 mm criteria
Wang et al. 2020^51^	576 Plans	Multiple	DNN	Gamma passing rate	54	Absolute prediction error = 1.76% at 3%/3 mm criteria
Kimura et al. 2020^52^	161 Beams	Prostate	CNN	Errors detection	145	3 Classes: Accuracy = 0.94

Abbreviations: ANN, artificial neural network; AUC, area under the receiver operating characteristics curve; CNN, convolutional neural network; DNN, deep neural network; MAE, mean absolute error; SVM, support vector machine.

Li et al.^45^ built a Poisson regression model with LASSO regularization to predict the gamma passing rates from complexity features of VMAT plans for patient‐specific QA measurements using a 2D array detector. The model achieved a prediction accuracy within 3.5% for 90% of the cases with 3%/3 mm gamma criteria. Ono et al.^46^ evaluated various ML models including regression tree analysis, multiple regression analysis, and ANN for gamma passing rate predictions using plan complexity features for patient‐specific QA measured using 3D array detector. Among the tested models on the same dataset, ANN had achieved the best prediction performance with a mean prediction error of −0.2%, followed by multiple regression analysis (mean prediction error of 0.5%), and lastly regression tree analysis (mean prediction error of 0.6%). Wall and Fontenot^48^ also studied several ML models including linear, SVMs, tree‐based (decision tree, random forest, AdaBoost, and gradient boosting), and ANN trained on plan complexity features for predicting VMAT QA gamma passing rate measured using a 2D array detector. The best performance was achieved by the SVM with a Mean Absolute Error (MAE) of 3.85%, followed by the gradient boosting model with a MAE of 3.94%, then random forest and AdaBoost models both with a MAE values <4%. Hirashima et al.^49^ studied tree‐based models using XGBoost for gamma passing rate prediction on VMAT plans trained on mixture of plan complexity and radiomics features with patient‐specific QA measured using 3D array detector. The models obtained a MAE of 4.2% in predicting the gamma passing rate at 2%/2 mm criteria. Tomori et al.^50^ examined a CNN model trained on features derived from dose distribution images to predict the gamma passing rates for patient‐specific QA measurements using a 2D array detector. The model achieved a prediction performance of MAE of 0.63%. Wang et al.^51^ studied a DNN model for gamma passing rate predictions using complexity features for VMAT QA measurements using 3D array detector. The system performance was better than a Poisson regression model tested on the same data set (mean prediction error of 1.76% vs. 2.10% at 3%/3 mm gamma criteria).

Granville et al.^47^ developed an SVM model trained on complexity features for classifying the VMAT patient‐specific QA measurements performed with 3D array detector. They used three error classification groups based on the median difference between the measured and calculated doses for each plan: (1) larger than 1%; (2) smaller than –1%; and (3) between −1% and 1%. The results showed that the prediction model achieved a macro‐average AUC of 0.88. This shows that it is possible to use ML models to classify the median dose deviations that would be measured for a particular VMAT patient‐specific QA. Kimura et al.^52^ studied a CNN model for the MLC positional error prediction by using features derived from dose difference maps with QA measurement performed using 3D detector device. The results showed that CNN model are capable of predicting the error types of the plans as error‐free, systematic error, or random error with an overall accuracy of 0.944. Carlson et al.^44^ inspected single and multiple linear regressions, random forest, and cubist to predict the MLC positional errors during VMAT delivery using log files data (leaf motion parameters) from multiple institutions. The study results showed that MLC positional errors are predictable with a maximum RMSE of 0.196 mm (cubist), 0.193 mm (linear regression), and 0.200 mm (random forest). The gamma passing rate results that were calculated by incorporating the predicted MLC positional deviations during the head and neck VMAT plan optimization improved with an average of 4.17%.

### 4.3 Comparison based on data set size

The data set size that is required to train ML‐based and DL‐based models for accurate prediction varies greatly, with DL models generally requiring larger dataset size. The large variations in the data set sizes (303–1620 data samples, Tables 1 and 2) that were used for training the different ML models make the comparison of the reviewed studies difficult. A small dataset size could lead to imperfect prediction results and wrong conclusions due to the high likelihood of model overfitting. Valdes et al.^33^ reported that about 200 data samples are required to train an ML‐based predictive model successfully for virtual patient‐specific QA for one Linac. If one model is intended to provide patient‐specific QA predictions for different Linacs at the same institution, more plans would be needed due to a higher variance of the data. As a result, most ML models trained with lower data samples need to be validated on a large‐scale dataset. Regardless of the prediction performance, ML and DL models that are trained on large datasets^38^, ^40^, ^47^, ^49^ provide more reliable results. The results reported by Carlson et al.,^44^ Osman et al.,^41^ and Chuang et al.^43^ used large data sets, ranging from hundred‐thousand to millions data samples, for training various ML models. Compared to ML, a DL model needs larger datasets to properly train and to optimize its hyper‐parameters.^53^ In the reviewed studies, some researchers used a dataset of relatively low number of samples (60–576 data samples, shown in Tables 1 and 2). This is not sufficient for proper training of DL models and is more likely to induce model overfitting. One widely used approach to overcome the problem of large‐scale data size requirement in DL is the transfer learning technique^29^ which allows training a DL model with limited amount of data. However, this approach was used only by Interian et al.^36^ in training CNNs on a dataset of 498 IMRT plans to predict the gamma passing rate results. More studies should be undertaken using transfer learning or data augmentation to validate their performance and ensure that there is no overfitting problem.

It is very difficult for a single institution to collect a large dataset of several thousand IMRT or VMAT plans with their QA data for model training. Balance of data samples within the training dataset is important to avoid bias of the model in the prediction results. If the training dataset contains only few types of treatment sites the dataset will be biased from the point of anatomical target shape. Preparing high‐quality data for building an accurate prediction model requires a dedicated supervised human effort to collect large data samples from the radiation oncology databases. In addition, major effort is required for labeling the data and handling the missing data. Therefore, multi‐institutional efforts to establish a large dataset or sharing data between institutions would be useful and effective to overcome the limited data availability. This would allow the comparison of the performance of the developed models on a unified dataset. Making the datasets publicly available would also be beneficial to researchers, in particular in developing DL‐based models which require large‐scale dataset from multiple institutions.

### 4.4 Comparison based on the predictive features

Various types of feature have been used for training the ML/DL models to predict the patient‐specific QA outcome results. These types of features include complexity metrics derived from the plans and machine‐related parameters, radiomics analysis and CNN‐features derived from images (e.g. dose/fluence maps), or the combination of these features. In order to eliminate unimportant features and reduce the complexity of the model, an adequate feature selection method should be implemented to identify and use the most important ones. This helps to simplify interpretation and visualization of the data, and improve the overall performance and robustness of the predictive model.^54^

Several studies applied features selection techniques to identify only predictive features hence minimizing the likelihood of model overfitting. The feature selection methods that were implemented include LASSO algorithm,^33^, ^34^ SVM recursive feature elimination algorithm,^40^, ^47^ forests of extremely randomized decision trees “Extra‐Trees” algorithm,^48^ mutual information algorithm,^48^ DL dropout technique,^35^, ^40^, ^50^, ^52^ linear regression algorithm,^48^ Wilcoxon rank‐sum test,^37^ random forest,^42^, ^49^ Pearson's correlation coefficient,^46^ and principal component analysis algorithm.^39^, ^45^ It is important to note that different feature selection methods applied to the same dataset may result in selecting different features. A summary of QA measurement device, feature selection methods, and the important predicative parameters is presented in Table 3.

**TABLE 3:** Summary of features used as inputs to ML/DL models for IMRT/VMAT QA outcome predictions.

Input features	QA technique	Feature selection technique	Important features	Reference
Plan complexity features	2D array detector	LASSO	4 features: MU factor, aperture score, irregularity factor, and fraction of the plan delivered at the corners of a 40 × 40 cm^2^ field	Valdes et al.^33^
	2D array detector and EPID	LASSO	7 features: irradiated area outline, Jaw position, fraction of the area receiving dose from penumbra, Duty cycle, irregularity factor, and others	Valdes et al.^34^
	Film dosimetry	Dropout technique	4 features: MU values, the PTV volume, the rectum volume, and the overlapping region volume	Tomori et al.^35^
	3D array detector	Pearson's correlation coefficient	28 features: plan complexity parameters (*n* = 18), machine type (*n* = 4), and photon beam energy (*n* = 6).	Ono et al.^46^
	3D array detector	SVM recursive feature elimination	30 features: Linac output, MU factor, total number of control points, and others	Granville et al.^47^
	2D array detector	Manually	54 features: MU value, union aperture area, plan area/irregularity/modulation, average leaf gap/dose rate/travel distance, modulation index of leaf speed/acceleration, and others	Li et al.^45^
	EPID	Manually	10 features: modulation complexity score, beam irregularity, MUs/control point in a beam, maximum of *x*–*y* jaw positions, edge metric, and others	Lam et al.^38^
	3D array detector	Manually	54 features: plan modulation‐complexity and delivery‐characteristics	Wang et al.^51^
	2D array detector	Extra‐trees, mutual information, and linear regression	100 features: aperture score, MU factor, edge metric, leaf gap/travel/motion, plan irregularity, plan modulation, and others	Wall and Fontenot^48^
	2D array detector	Manually	6 features: leaf position, instantaneous velocity, movement away/toward the center, leaf movement status, the control point number, and the leaf bank	Carlson et al.^44^
	EPID	Manually	14 features: leaf previous/current/next positions, dose fraction, gantry angle, leaf speed/acceleration, leaf gap, leaf movement status, and others	Osman et al.^41^
	EPID	Manually	7 features: leaf velocity, acceleration, control point, dose rate, gravity vector, gantry velocity (VMAT), and gantry acceleration (VMAT)	Chuang et al.^43^
Radiomics features	EPID	Wilcoxon rank‐sum Test	13 features: radiomics features (size zone metric) and intensity histogram metrics derived from gamma map images	Wootton et al.^37^
	2D array detector	Dropout technique	CNN features derived from fluence map images	Interian et al.^36^
	2D array detector	Dropout technique	CNN features derived from dose distribution images	Tomori et al.^50^
	EPID	SVM recursive feature elimination	11 features: radiomics features e.g. contrast, uniformity, zone entropy, and others	Ma et al.^40^
	3D array detector	Dropout technique	CNN features derived from dose difference or gamma map images	Kimura et al.^52^
	EPID	Random forest regression	11 features: radiomics intensity histogram features and the texture features derived from fluence difference map images	Sakai et al ^42^
Combined features	EPID	Principal component analysis, and triplet networks	81 features: 17 texture features (hand‐crafted) from intensity histograms and size zone matrices) and 64 CNN features derived from gamma map images	Nyflot et al.^39^
	3D array detector	Random forest	502 features: plan complexity features (e.g. aperture area/perimeter/irregularity, and others), radiomics features (shape, statistical information, and texture features), and clinical parameters (treatment site/machine, beam energy, and dose calculation algorithm)	Hirashima et al.^49^

Abbreviations: CNN, convolutional neural network; EPID, electronic portal imaging device; LASSO, Least Absolute Shrinkage and Selection Operator; MU, monitor unit; PTV, planning target volume; SVM, support vector machine; VMAT, volumetric‐arc radiation therapy.

#### 4.4.1 Complexity features

Plan complexity features are defined as quantitative metrics for the MLCs characteristics such as shape, aperture size, travel leaf motion, speed, and complexity score.^55^ It has been reported in the literature that complexity features such as aperture area, leaf gaps, and jaw positions are predictive of delivery accuracy.^55^, ^56^ Thus, these complexity metrics with other machine‐related parameters (e.g. machine type and beam energy) and clinical parameters (e.g. treatment site) can be used as model input features. Various ML and DL models were trained to map the plan complexity features alone or with other dosimetric features.^33^, ^34^, ^35^, ^38^, ^41^, ^43^, ^44^, ^45^, ^46^, ^47^, ^48^, ^51^ Complexity metrics can be related to the QA outcome results, offering a troubleshooting method in case a plan fails the QA. Complexity metrics identified as important features include MU value,^34^, ^35^, ^38^, ^45^, ^47^, ^48^ beam irregularity factor,^34^, ^38^, ^45^, ^48^ aperture size/area,^34^, ^38^, ^45^, ^46^, ^48^, ^49^ and others (Table 3).

The dependency of prediction models on specific treatment machine, the QA device (2D/3D diode/ion chamber array, film, or EPID), the anatomic treatment site, the beam energy, and the dose calculation algorithm (e.g. Acuros XB, CCC, AAA, etc.) were investigated. The effect of different characteristics of the measurement devices from different manufactures on the QA outcome prediction model performance was assessed by Valdes et al..^34^ The authors trained a Poisson regression model on complexity features for IMRT QA predictions of gamma passing rate with measurements performed using a 2D diode array. Then, to generalize their model, they tested it on another institution dataset with QA measurements performed using EPID. The results indicated that ML/DL models trained on a single institution dataset may be generally applicable to other clinics, regardless of the differences in Treatment Planning Systems (TPSs), Linacs, and measurement devices. Similarly, treatment site dependency was evaluated^45^, ^48^, ^49^ by training an ML/DL model on a dataset consisting of different cancer sites. Wall and Fontenot^48^ and Hirashima et al.^49^ reported that the treatment site has dependency with the gamma passing rate predictions. As results, specific models should be built for different anatomic treatment sites. Energy dependency was also studied by Valdes et al.,^33^ and it was found to be an important feature affecting the model predictive performance. This is because different beam energies have different dose profile characteristics. For dosimetrically matched Linacs (e.g. dosimetric leaf gap and MLC leakage), variations are lower and a prediction model can be trained on a dataset from the matched Linacs.^38^

#### 4.4.2 Radiomics analysis and CNN features

*Feature‐engineered radiomics method* involves hand‐crafting feature extractions and requires manual feature engineering. Radiomics features capture characteristic patterns in the imaging data, including shape (e.g. sphericity), first‐order, second‐order, and higher‐order statistical determinants and image‐based (e.g. fractals) features. Limited studies used radiomics texture features extracted from dose/fluence distribution^40^, ^42^, ^49^ or gamma map images^37^, ^39^ to train an ML/DL model for patient‐specific QA outcome predictions. Features extracted from dose difference maps^42^ were found to be more predictive and provide more accurate MLC positional errors than those derived from gamma maps.^37^ In contrast with complexity metrics, radiomics features extracted from dose/fluence distributions or gamma maps have a limitation which is the lack of direct relation between the features and the QA results. Radiomics features that were found to be significantly predictive for IMRT/VMAT QA outcome include gray level co‐occurrence matrix^37^, ^49^ (Table 3).

*Non‐feature‐engineered radiomics methods*, in contrast with the hand‐crafted radiomics features based approach, involves automatic feature extractions from the input images using DL without human expert supervision. This auto‐extraction is better and more flexible than the tedious process in the hand‐crafted method. DL models perform the learning of data with multiple levels of abstraction. For instance, the front‐end layers in the CNN encode low‐level features in the image common to most computer vision applications, whereas the subsequent layers learn high‐level features which are more application‐specific. Few studies^36^, ^39^, ^50^, ^52^ implemented feature‐less approach in training ML/DL models for patient‐specific IMRT/VMAT QA outcome predictions. CNN features extracted from dose distribution,^50^ dose difference,^52^ fluence map,^36^ and gamma map images^39^, ^52^ were applied to train various ML/DL models. Results have shown that using the feature‐less approach for training the models provide comparable or higher prediction performance compared to using radiomics features derived by the hand‐crafting approach^39^ or complexity metrics.^36^ Although feature‐less approach embedded in DL models has an advantage of providing more freedom and flexibility in feature extraction, it is challenging to interpret the extracted features.

#### 4.4.3 Combined (hybrid) features

Training a model using hybrid features such as plan complexity metrics, radiomics features, and clinical features for IMRT/VMAT QA predictions is expected to provide improved accuracy compared to using only one type of features. Combining different types of features provides comprehensive information that could improve the predictions. Two studies^39^, ^49^ have investigated the potential of using different types of features for IMRT/VMAT QA outcome predictions (Table 3). CNN‐extracted features combined with radiomic features derived from gamma map images was used by^39^ to train different ML model to predict MLC errors during IMRT delivery. This study reported that ML models trained with CNN‐features exhibited higher performance than models trained on radiomics features. This is due to the fact that feature‐less method allows extracting higher number of features compared to hand‐crafted approach. Hence, more patterns in the input images are recognized and better prediction accuracy is achieved. A mixture of complexity metrics, radiomics features, and clinical parameters were used by Hirashima et al.^49^ to train tree‐based ML models for VMAT QA gamma passing rate outcome predations. This study showed that using a combined set of features improves the model prediction accuracy.

### 4.5 Comparison based on the QA outcome prediction approach

**FIGURE 2:**
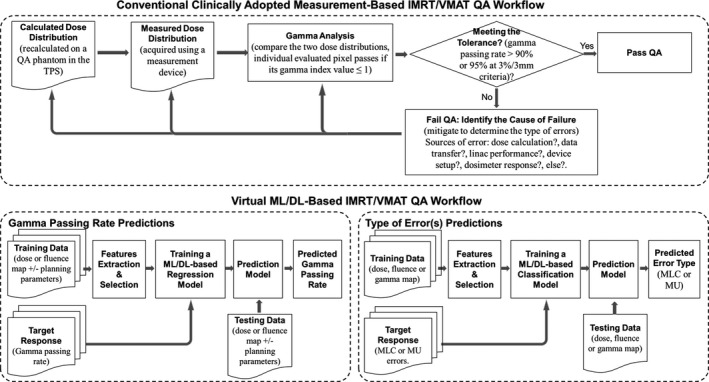
A typical flow diagram of patient‐specific intensity modulated radiation therapy/volumetric‐arc radiation therapy (IMRT/VMAT) quality assurance (QA). (Top) current clinically adopted QA based‐measurement approach, (Bottom) machine learning/deep learning (ML/DL)‐based approach for predicting the gamma passing rate results and detecting or identifying the types of QA errors for failing plans

Patient‐specific QA outcome predictions with ML/DL in IMRT and VMAT could be categorized into two approaches. The first one deals with directly predicting the gamma passing rate results. The second approach focuses on detecting errors that are associated with the delivery and cannot be discovered with the gamma passing rate metric. A block diagram showing the workflow of ML/DL models implementation in predicting patient‐specific QA of IMRT/VMAT delivery is presented in Figure 2. The workflow can be generally described as follows: (1) acquire the IMRT/VMAT plans and QA measurement data; (2) derive and extract the features and parameters from the data then select the most predictive ones; (3) train and validate a ML/DL model to learn mapping the selected input parameters and features to gamma passing rate or errors detection; and (4) use the trained model to predict the gamma passing rate results or identify errors during IMRT/VMAT delivery.

#### 4.5.1 Predicting gamma passing rate results

Directly predicting the gamma passing rate results with ML/DL models for patient‐specific IMRT/VMAT QA were studied by numerous investigators. Gamma analysis is widely accepted as a clinical metric to evaluate the dose distributions, and a passed/failed IMRT/VMAT plan is determined according to the value of the gamma passing rate. It provides quantitative analysis at the pixel level and gives information about the number of pixels that passed the gamma analysis criterion. IMRT or VMAT plan passes the QA if the gamma passing rate is ≥95% at global 3%/3 mm criteria (action needed if the gamma passing rate <90%).^1^, ^57^ The prediction of the gamma passing rate with ML/DL models can be treated as a regression task to estimate the percentage of the evaluated dose (or fluence) points that pass the gamma criteria (e.g. 3%/3mm, 2%/3mm, 2%/2mm, etc.). Based on this predicted value, the plan is determined to be passing or failing the QA according to an acceptable threshold (e.g. 95% or 90%) which depends on the applied gamma criteria. Regression models have the advantage of providing more quantitative information. Directly predicting the QA outcome (e.g. passing or failing results) can also be treated as a classification task.

Eleven studies have implemented ML/DL models for IMRT/VMAT QA gamma passing rate predictions (Tables 1 and 2) including Poisson regression,^33^, ^34^, ^45^ XGBoost,^38^, ^49^ AdaBoost,^38^, ^48^ random forest,^38^, ^48^ regression tree analysis,^46^ multiple regression analysis,^48^ and ANN,^46^, ^48^, SVMs,^48^ decision tree,^48^ gradient boosting,^48^ CNNs,^35^, ^36^, ^50^ and DNNs.^51^ All the models were trained on datasets consisting of multiple treatment sites, except the study by Tomori et al.^35^ where the dataset was site‐specific (prostate). In these studies, three measurement techniques were routinely used for IMRT/VMAT QA: portal dosimetry using EPID, 2D/3D array detectors, and film dosimetry using EBT Gafchromic film as shown in Table 3. ML and DL models applied to map the input features to gamma passing rates for patient‐specific QA in IMRT and VMAT have shown the potential for accurate predictions. These predictive models can help guide the plan optimization process to avoid solutions which are likely to result in lower gamma passing rate during QA.

#### 4.5.2 Predicting errors during delivery

Although the gamma analysis is often used to determine if an IMRT/VMAT plan can be accurately delivered, it has some limitations as well. One of these limitations is that it does not reveal clinically significant errors such as random MLC errors.^6^, ^58^, ^59^ Another limitation is that it is often difficult for clinical physicists to identify and rectify the cause(s) of errors.^6^ This is because patient‐specific IMRT/VMAT QA measurement can be affected by several potential sources of error including dose calculation, data transfer, Linac performance, device setup, and dosimeter response.^3^ Some studies presented potential solutions by applying ML/DL for detecting and identifying these errors for more effective patient‐specific QA. Error detection and identification with the ML/DL models is a classification task and has the advantages of providing a quick, unambiguous, and actionable result. However, quantifying the error (e.g. MLC positional errors) is a regression problem to determine the value of the deviation.

As presented in Tables 1 and 2, nine studies investigated the capability of ML/DL models for errors detection. ML and DL algorithms for this task include logistic regression,^37^, ^42^ linear regression,^43^, ^44^ random forest,^40^, ^42^, ^44^ cubist,^44^ SVMs,^39^, ^40^, ^42^, ^47^ ANN,^39^, ^41^ decision tree,^39^, ^42^, ^43^ KNN,^39^, ^42^ discriminant analysis,^40^ CNN,^52^ and ensemble of tree‐based (bagged and boosted).^43^ Detectability of error category such as free of error, random MLC error, systematic MLC error, transmission factor error, dosimetric leaf gap error, or MU/machine output variations using ML/DL models were investigated.^37^, ^39^, ^40^, ^42^, ^47^, ^52^ Three studies^41^, ^43^, ^44^ in the literature focused on utilizing the ML algorithms to predict the individual leaves positional deviations using the log file data. These predicted positional deviations of all leaves are then incorporated into the TPS to be taken into account during the plan optimization process. This procedure can significantly help in enhancing the gamma passing rates results of patient‐specific QA.

The reviewed studies for errors detection and identification models have shown that MLC positional errors, MLC modeling parameters, and MU/machine output variations could be predicted with ML/DL models. Extending the predicted types of errors to include other errors such as setup errors, detector calibration errors, beam modeling errors, and dose rate errors would provide the physicists valuable information to mitigate these errors.

### 4.6 Comparison based on models validation

Model validation is very important step after the training of ML and DL models, where the model performance is tested on a validation dataset. The validation is required for the reliability of the developed prediction models. Reporting the model performance on a dataset that was not seen by the model during the training warrants the model accuracy and applicability to real‐world data.

In this review, model validation was performed using the *k*‐fold cross‐validation^33^, ^35^, ^36^, ^38^, ^40^, ^42^, ^45^, ^47^, ^48^, ^49^, ^50^, ^51^, ^52^ and the hold‐out validation^37^, ^39^, ^41^, ^43^, ^44^, ^46^ techniques. In *k*‐fold cross‐validation, the dataset is equally partitioned into *k* subparts or folds. Of the *k*‐folds or groups, for each iteration, one group is selected as validation data, and the remaining (*k* − 1) groups are selected as training data. The process is repeated for *k* times until each group is treated as validation and remaining as training data. The cross‐validation technique could be very useful when there is not enough data, hence the available data are randomly divided into a development set (training and cross‐validation) and a testing set. In the other hand, the hold‐out validation technique involves separating part of the data as a subset for independent validation. Some models^37^, ^39^, ^43^, ^46^ were trained on a training set and their performances were validated on a testing set. In some other studies,^41^, ^44^ the dataset was into three separate sub‐sets for training, validation (fine‐tune the model), and testing. In one study,^34^ the model validation was performed on a test set from another institution that has different data characteristics from the training dataset. This validation technique is suitable for large data size and reduces the model bias and dependence on the training data.

### 4.7 Comparison based on models performance

Our review shows that ML and DL models have demonstrated a high degree of prediction accuracy for patient‐specific QA in IMRT and VMAT. There are large differences in model performances. Model performance may be limited by characteristics of the underlying data, particularly the unique and specific combination of technologies and clinical parameters used to generate treatment plans and perform QA. Each model was trained on a different dataset including single institution dataset, multi‐institution dataset, single treatment site, multiple sites, different measurement devices, different Linacs, etc. Hence, the models give different prediction results. These variations in the type of dataset make the direct comparison of the performance of these models difficult. However, some comparisons could still be made to provide some quantitative and qualitative information.

The accuracies achieved by the ML/DL models for the prediction of gamma passing rate results have shown a prediction accuracy within 3%. These included Poisson regression with 3.0% error,^33^ AdaBoost with 3.0% errors,^38^ random forest with 3.0% errors,^38^ DNN with 1.8% mean prediction error,^51^ CNN with 1.1% error,^35^ ANN with less than 1.0% mean prediction error,^46^ regression tree analysis with less than 1.0% mean prediction errors,^46^ and multiple regression analysis with less than 1.0% mean prediction error.^46^ Here, we emphasize on reporting multiple evaluation metrics for each developed model so that meaningful comparison between models can be performed. When a DL and an ML model were evaluated on the same dataset, the DNN model outperformed Poisson regression ML model in gamma passing rate predictions with absolute prediction error of 1.8% versus 2.1%.^51^ Similarly, when various ML models were evaluated on the same dataset, models that have shown the highest performance regardless of their prediction accuracy are regression tree analysis,^46^ AdaBoost and random forest,^38^ and SVM.^48^ As demonstrated from the analyzed results, prediction accuracy within 3% can be achieved for IMRT/VMAT patient‐specific QA gamma passing rate results. Most of the reported results in the literature were for QA data analyzed with 3%/3mm gamma criteria which is not stringent. More stringent gamma criteria such as 2%/2 mm was found to be more sensitive and exhibited dramatically improved clinically relevant errors detection.^6^ However, large‐scale QA dataset with this gamma criteria is not available currently, and the very limited available dataset had a high inherent noise for accurate modeling.^34^, ^35^

Machine learning/deep learning models detection of different types of errors for IMRT/VMAT QA were evaluated. Deep learning has shown the potential for accurate error detection for patient‐specific QA.^52^ It was not surprising that the CNN model achieves the highest performance in this classification task among all reported models with an overall accuracy of 0.94 in predicting three classes of MLCs positioning errors (error‐free, random, and systematic).^52^ The CNNs excel at tasks involving analysis of images such as gamma or fluence/dose maps. This result also indicates that features derived from dose difference map directly reflect the MLC positional errors. However, this high prediction accuracy of DL models is compromised with low model interpretability. In predicting every single error such as error‐free versus MLC error, error‐free versus dosimetric leaf gap error, the SVM ML model achieved the best performance.^42^ Various studies^39^, ^40^, ^42^ showed the superior performance of SVM models for errors detection when evaluated with different ML models on the same dataset. The reported results show that ML/DL has the potential to improve error detection and provide decision support for classification of root causes of QA failure. Larger datasets from multiple institutions are required to re‐train the developed models as well as to ensure the models generalizability to other clinics. However, curating a large library of QA images with annotated error types remains a challenge.

Overall, patient‐specific QA gamma passing rate results could be accurately predicted within 3% accuracy using ML/DL models for IMRT and VMAT. Similarly, errors associated with failed QA plans could also be identified with high degree of accuracy. Reporting just a single evaluation metric such as AUC or MAE is not sufficient for fair and meaningful comparison of the model performance results. Establishing a consensus regarding preferred metrics for the performance of these models is recommended. The comparison made here is for different models using different datasets. A more objective comparison might be achieved if all the models were tested on a common open‐source dataset. Due to limitation of availability of a large dataset for each treatment site, the reported results for the most predictive models were trained using multiple anatomic treatment sites data such as head and neck, prostate, lungs, abdomen, pelvis, etc. Only two reviewed studies used a dataset of one anatomic site which is prostate.^35^, ^52^ These two site‐specific models (CNNs) achieved higher performance compared to other models. The degree of complexity in an IMRT or VMAT plan is likely to depend on the treated site, hence the accuracy of the reported models trained on data of multiple sites could be improved by building an specific model for each anatomical treatment site.^34^ To achieve this target, more data for each treatment site is required. These prediction models were trained on different sets of relatively small data size. The reported performance could further be improved as larger datasets from different institutions become available.

### 4.8 Clinical applicability

Based on the patient‐specific IMRT/VMAT QA results, a clinical decision is made whether a plan is accurate and safe for patient treatment. When ML/DL algorithms are used for development of a model for a clinical task such as IMRT/VMAT QA outcome predictions, then the model should be accurate and interpretable thereby providing confidence for clinical utility.

Presently, the patient‐specific IMRT/VMAT QA gamma passing rate results could be predicted with ML and DL models within 3% errors. This accuracy was achieved by various DL and ML models, which makes these models promising for clinical application. However, it should be highlighted that the reported performances of these models lack the generalizability because they were trained on institution‐specific dataset. The reported accuracies might degrade when the models are tested in other clinics that use different TPSs, delivery machines, and measurement devices. These models depend on the combination of technologies used which can vary from one institution to another. Requiring each institution to train or tweak a ML or DL model to make it suitable for their particular technologies is probably unacceptable.

The overall evaluation of a model should consider assessing the model prediction accuracy, robustness, and interpretably for clinical applicability. Generally, as the model prediction accuracy increases its interpretability becomes more difficult. Tree‐based ML models have easy (decision trees) to moderate (random forests) interpretability and prediction accuracy. Consequently, one can understand each feature's contribution to the prediction results. The performance of a model can be greatly improved by using an ensemble through bagging (random forests) and boosting (e.g. adaptive boosting). Linear regressions are the easiest ML models to interpret but their prediction accuracy is lower with higher possibility of underfitting the data. In contrast, deep neural networks are frequently surpassing the others models performances^36^, ^51^ but they suffer from being hard to interpret with more likelihood of overfitting. Although the overfitting problem could be minimized by applying a proper regularization technique.

If a facility wanted to verify the accuracy of a trained ML/DL model for their Linac for IMRT/VMAT QA, they should perform patient‐specific QA measurements for a number of plans and compare the gamma passing rate values with the predicted ones. A comparison of the gamma passing rate for a specific plan with other institutions could also be valuable to compare the accuracy of their commissioning and TPS data. This will require building models for each combination of equipment or methodology for pretreatment verification used by different institutions. For errors detection of IMRT/VMAT plans that fail the QA, development of an ML/DL model that could accurately identify all potential sources of errors is required for effective clinical application. Therefore, this task is an ongoing field of research and it is presently far from the clinical usage.

To our knowledge, the deployment of the developed patient‐specific QA prediction models into clinical practice is still lagging due to of some challenges. For instance, standardization guidelines are needed for data content. Another challenge is the availability of large dataset to confirm the model stability, consistency, and generalizability. Moreover, model verification and comprehensive clinical validation should be done to ensure that the model is robust. Patient‐specific QA outcome prediction offers no help if the outcome predicted does not actually correlate with clinical results.^60^

All the reviewed papers were published within the last 5 years, making it a new area of interest with high potential for future research and development to achieve clinical acceptance and integration. Proper understanding of the mathematical principles behind the chosen algorithm, identifying the model strengths and weakness, and interpreting the predicted results knowledge are extremely important and should be adequately transferred to the clinicians. Full understanding of these aspects would greatly help the physicists to effectively perform QA and gain confidence in the QA prediction models. The physicist needs to ensure the quality and safety of the virtual QA task before it can be integrated into clinical practice. In order to achieve clinical applicability, close and effective collaboration among the clinicians, mathematicians, computer scientists, and data scientists is required.

### 4.9 Potential clinical impact

An accurate prediction ML/DL model is practical and can help clinical workers reduce the burden of performing measurements for patient‐specific QA. This is important for resource‐limited countries where machine time is typically not available for IMRT/VMAT QA for all patients, or for adaptive radiation therapy techniques which involve changing the plan while the patient is on the table. It would enable the dosimetrists to know in advance whether a plan would pass or fail QA as well as the potential root cause(s) of failure immediately after the treatment plan is created. A prior information regarding the deliverability of plans during the optimization stage could provide many benefits that include minimizing the time wasted in performing QA measurement. In this way, failing plans could be potentially eliminated and a treatment delay due to the failure of IMRT QA can be avoided. Virtual IMRT/VMAT QA could be performed during the planning process, thus avoiding QA measurements. It also helps to achieve a fully automated patient‐specific QA tool for treatment plan verification.

## 5 CONCLUSIONS, RECOMMENDATIONS, AND FUTURE DIRECTIONS

A comprehensive review on various successfully developed ML/DL‐based models for patient‐specific IMRT/VMAT QA outcome predictions was performed. Patient‐specific QA gamma passing rate can be predicted with errors within 3.0% with various ML/DL models for different treatment machines and different underlying datasets. Types of errors for an IMRT/VMAT plan that failed the QA have been identified using ML and DL models with promising accuracy. Developing errors detection ML/DL models would provide more effective, efficient, and robust tool for patient‐specific QA. The potential clinical benefits of this virtual patient‐specific QA tool lie in reducing the time of actual measurements and the physicist's workload hence making the treatment process more efficient.

In order to be integrated into clinical practice, the QA prediction model performances need to be validated on large‐scale multi‐institution datasets to evaluate their generalizability to different clinics. Treatment site‐specific models are highly recommended whenever the dataset size permits. We recommend training ML and DL models on a large‐scale dataset from multiple institutions. As this review showed that DL has overall higher prediction accuracy compared to ML models. We recommend the use of pre‐trained DL models on the QA data as a base to train the models via transfer learning and not to train the models from scratch at every clinic to get improved performance.

The future directions of research in this subject would be in training the ML/DL models on anatomical region‐specific IMRT/VMAT plans (e.g. brain and head and neck, chest, abdomen, and pelvic regions) to improve the current reported performance results of patient‐specific QA. Future studies should also focus on improving the model prediction accuracy. For error detection, extension to additional errors associated with QA failure such as machine‐related errors, setup errors, detector calibration errors, should be adopted.

## AUTHOR CONTRIBUTION

Alexander F. I. Osman involved in conceptualization, methodology, investigation, writing the original draft, and visualization. Nabil M. Maalej involved in conceptualization, reviewing, and editing the original draft.
